# *RAS* Mutations in AUS/FLUS Cytology: Does it Have an Additional Role in BRAF^V600E^ Mutation-Negative Nodules?

**DOI:** 10.1097/MD.0000000000001084

**Published:** 2015-07-13

**Authors:** Jung Hyun Yoon, Hyeong Ju Kwon, Hye Sun Lee, Eun-Kyung Kim, Hee Jung Moon, Jin Young Kwak

**Affiliations:** Department of Radiology, Severance Hospital, Research Institute of Radiological Science (JHY, E-KK, HJM, JYK); Department of Pathology, Yonsei University, College of Medicine (HJK); Department of Pathology, Yonsei University, Wonju College of Medicine (HJK); and Biostastistics Collaboration Unit, Medical Research Center, Yonsei University, College of Medicine (HSL), Seoul, South Korea.

## Abstract

The object of this study is to evaluate the additional role of *RAS* mutation in detecting thyroid malignancy among BRAF^V600E^ mutation-negative nodules diagnosed as atypia of undetermined significance/follicular lesion of undetermined significance (AUS/FLUS) on cytology.

From December 2009 to December 2011, 202 BRAF^V600E^ mutation-negative thyroid nodules diagnosed as AUS/FLUS cytology in 201 patients were included in this study. *RAS* mutation analysis was performed using residual material from ultrasonography-guided fine needle aspiration (US-FNA) cytology testing for *K-RAS*, *N-RAS*, and *H-RAS* codons 12/13 and 61 point mutations. The authors evaluated the association between *RAS* mutation status and cytopathologic characteristics.

Of the 202 BRAF^V600E^ mutation-negative thyroid nodules with AUS/FLUS cytology, 4 were considered insufficient for mutation analysis. Of the 198 thyroid nodules, 148 (74.7%) were confirmed as benign and 50 (25.3%) as malignant. Thirty-one (15.7%) of the 198 thyroid nodules were positive for any *RAS* mutation, 4 positive for *K-RAS* 12/13, 26 for *N-RAS* 61, and 1 positive for *H-RAS* 61. Seven (22.6%) of the *RAS* mutation positive nodules were malignant, 1 with *K-RAS* 12/13, 6 with *N-RAS* 61. Twenty-four (77.4%) of the 31 nodules positive for *K-RAS* 12/13 (N = 3), *N-RAS* 61 (N = 20), or *H-RAS* 61 (N = 1) mutations were proven benign. None of the 198 thyroid nodules were positive for *K-RAS* 61, *N-RAS* 12/13, or *H-RAS* 12/13 mutations.

*N-RAS* 61 mutation is the most common mutation detected among BRAF^V600E^ mutation-negative nodules with AUS/FLUS cytology. *RAS* mutation has limited value in predicting malignancy among BRAF^V600E^ mutation-negative thyroid nodules with AUS/FLUS cytology and further, investigation is anticipated to evaluate the true role of *RAS* mutation in thyroid malignancy.

## INTRODUCTION

Although ultrasonography-guided fine needle aspiration (US-FNA) is considered the gold standard showing excellent diagnostic performances in the diagnosis of thyroid nodules,^1–4^ 1 of the main flaws for this diagnostic method is “indeterminate” categories,^5^ including atypia of undetermined significance/follicular lesion of undetermined significance (AUS/FLUS), follicular neoplasm or suspicious for follicular neoplasm, and suspicious for malignancy categories according to the recently proposed Bethesda system for reporting thyroid cytopathology.^6^ Considering the broad range of malignancy rates among these categories, 5% to 75%, cytology results of these categories warrant further invasive investigations such as repeat US-FNA or diagnostic lobectomy,^5–7^ among which approximately 66% will eventually be proven benign.^8,9^ Atypia of undetermined significance/follicular lesion of undetermined significance in particular, is a heterogeneous cytologic category, including a broad disease spectrum, from patients with air-drying artifact, low cellularity, or obscuring elements to architectural or nuclear atypia.^6^ Malignancy risk for this category has a broad range, reported from 6% to 42.1%,^10–15^ with management guidelines varying among reports or institutions accordingly. At present, improving the accuracy of preoperative diagnosis for patients with this ambiguous cytologic category is critical in patient management.

With the rapid evolution in molecular genetics, molecular analysis has been reported and proposed as an effective adjunct to cytologic evaluation in the differential diagnosis of thyroid nodules, especially those with nondiagnostic or indeterminate cytology.^16–19^ BRAF^V600E^ mutation in particular, is present in 29% to 84% of papillary thyroid carcinoma (PTC),^16,20–23^ showing high specificity up to 100% and is potentially a useful marker in predicting PTC in clinical practice. Several recent studies have applied BRAF^V600E^ mutation analysis in AUS/FLUS nodules,^11,24,25^ and although this additional method was helpful in stratifying nodules among this cytologic category, approximately 39.7% to 70.8% of thyroid nodules negative for BRAF^V600E^ mutation proved to be malignant.^11,24^*RAS* mutations are the second most common genetic alteration seen in thyroid cancers,^26,27^ and the most common genetic alteration seen in indeterminate thyroid nodules,^28^ but how it helps in detecting malignancy among AUS/FLUS nodules, especially in those negative for BRAF^V600E^ mutation, has not been clarified.

In this study, we evaluated the usefulness of *RAS* mutations when used as an adjunct for definitive diagnosis of thyroid nodules with AUS/FLUS cytology, which are negative for BRAF^V600E^ mutation.

## METHODS

This retrospective study has been approved by the Institutional Review Board (IRB) of Severance Hospital, Seoul, South Korea. Neither patient approval nor informed consent was required for review of medical records, cytology specimen, or US images. Signed informed consent was obtained from all patients before US-FNA or surgical procedures.

### Patients

The Bethesda system has been used in reporting thyroid cytopathology starting from December 2009 at our institution (a referral center), and since then, this reporting system has been consistently used. We performed a retrospective review of our institutional database for all thyroid US-FNAs performed on nodules measuring larger than 5 mm during the period from December 2009 to December 2011, and 13,456 US-FNAs had been performed during this period. Among them, approximately 725 (5.4%) nodules had been diagnosed as AUS/FLUS on US-FNA cytology. BRAF^V600E^ mutation analysis was performed in 300 (41.4%) nodules, of which 262 (87.3%) nodules had negative results. Thyroid nodules fulfilling the following inclusion criteria were included in this study: nodules confirmed with surgery or nodules that had been diagnosed as benign or malignancy on follow-up US-FNA. Finally, 202 nodules in 201 patients were included in this study. Mean age of the 201 patients was 58.5 ± 11.8 years (range, 18–76 years). Mean size of the 202 thyroid nodules were 19.0 ± 11.4 mm (range, 5–61 mm).

### US and US-FNA Procedures

Real-time US and US-FNA procedures were performed by 1 of 14 board-certified radiologists (3 faculty, 11 fellows, 1–15 years of experience) using a 5- to 12 MHz linear array transducer (iU22; Philips Medical Systems, Bothell, WA). Fine needle aspiration was performed on thyroid nodules exhibiting suspicious US features or at the largest mass among multiple benign-appearing nodules.

Ultrasonography-guided fine needle aspiration was performed with a 23-gauge needle connected to a 2 mL disposable syringe, using a freehand technique. Aspiration was performed at least twice for each thyroid nodule, during which aspirated material was expelled, smeared on glass slides, and placed immediately in 95% ethanol for Papanicolaou staining. The syringe used for aspiration was rinsed in normal saline for cell block processing. One of 5 cytopathologists specializing in thyroid cytopathology was involved in slide interpretation. Cytopathologists were not on-site during procedures, and additional immunohistochemical staining was performed on the cytopathologist's request. Since December 2009, cytology reports of our institution were based on the Bethesda system for reporting thyroid cytopathology.^6^

### BRAF^V600E^ Mutation Analysis

Additional BRAF^V600E^ mutation analysis was performed when the referring clinicians requested them, or when it seemed relevant to providing definitive diagnosis of thyroid nodules exhibiting suspicious US features. Aspiration was performed once more for BRAF^V600E^ mutation analysis after cytology slide preparations, which were rinsed in 1 mL of normal saline.

Dual priming oligonucleotide-based multiplex polymerase chain reaction analysis (DPO-PCR) was used for BRAFV600E mutation analysis from May 2008 to November 2011,^29^ and real-time PCR was used from November 2011 to the current date, according to the methods reported in prior studies.^29,30^

### *RAS* Mutation Analysis

*RAS* mutation analysis was performed at *N-RAS*, *H-RAS*, and *K-RAS* codon 12/13, and 61. Atypical cells of interest were isolated from US-FNA cytology smear slides, and DNA was extracted with QIAamp DNA mini kit (Qiagen, Germany) for PCR. A specific primer for exon 2 for amplification of codon 12/13 mutations and a primer for exon 4 for amplification of codon 61 mutations of the *N-RAS*, *H-RAS*, and *K-RAS* genes has been prepared for PCR reaction. The PCR reaction medium consisted of 2 μl DNA, 1X Real Helix^TM^ qPCR mixture (Nanohelix, Korea), and optimized concentrations (10 nM) of primers in a final volume of 20 μl. Polymerase chain reaction was performed under the following cycling conditions: predenaturation at 95°C for 10 minutes, 45 cycles at 95 °C for 30 seconds, 60°C for 30 seconds, and 72°C for 30 seconds, followed by final extension at 72°C for 5 minutes.

For sequencing analysis, the PCR product was purified, and single-strand reaction was carried out with each sequencing primer sets and BigDye® Terminator v3.1 Cycle Sequencing Kit (ABI, CA). The DNA sequence was obtained with an ABI PRISM 3730xl DNA Analyzer (ABI), and analyzed with the Sequencing Analysis 5.1.1 software.

### Data and Statistical Analysis

Histopathologic results from surgery and US-FNA were used as the standard reference. Nodules with benign cytology results on follow-up US-FNA, which had been followed with US examinations performed at an interval of more than 12 months, also showing stable or decreased size were considered benign. Nodules diagnosed as malignant on US-FNA but had not undergone surgery were considered malignant.

Independent 2-sample *t*-test was used in comparison of continuous variables. Chi-square test or Fisher exact test was used in comparison of categorical variables. All tests were 2-sided, and *P* values of less than 0.05 were considered to have statistical significance. Analyses were performed using SAS (version 9.2, SAS Inc., Cary, NC).

## RESULTS

Of the 202 BRAF^V600E^ mutation-negative thyroid nodules with AUS/FLUS cytology, 4 were found to have insufficient amount of isolated nucleic acid for mutation analysis. The remaining 198 thyroid nodules had undergone *RAS* mutation analysis. Of the 198 thyroid nodules, 148 (74.7%) were confirmed as benign, and 50 (25.3%) as malignant. Mean age of the patients with malignant nodules did not show significant differences to patients with benign nodules, 45.5 ± 10.9 years to 49.1 ± 11.8 years (*P* = 0.06). Mean size of the malignant nodules were significantly smaller than benign ones, 12.2 ± 10.3 mm to 16.2 ± 11.3 mm (*P* = 0.03).

Sixty-two patients with 62 (31.3%) thyroid nodules had undergone surgery; 32 were confirmed as classical type (PTC), 12 as PTC-follicular variant (FV-PTC), 9 as adenomatous hyperplasia, 3 as follicular adenoma, 2 as lymphocytic thyroiditis, 1 as medullary carcinoma, 2 as Hürthle cell adenoma, and 1 as Hürthle cell carcinoma, minimally invasive. One hundred thirty-six (69.7%) nodules in 135 patients had undergone follow-up US-FNA for diagnosis, and among them, 113 had benign cytology and 3 had malignant results on follow-up US-FNA. All the nodules with nondiagnostic (N = 6) and AUS/FLUS (N = 14) cytology on follow-up US-FNA had shown benign cytology results on third US-FNA.

Thirty-one (15.7%) of the 198 thyroid nodules were positive for *RAS* mutation, of which 7 (22.6%) were proven as malignant. Table 1 summarizes the *RAS* mutations to final pathology of the thyroid nodules included in this study. Of the 198 thyroid nodules, 4 were positive for *K-RAS* 12/13, 26 were positive for *N-RAS* 61, and 1 was positive for *H-RAS* 61. Of the 4 nodules positive for *K-RAS* 12/13, 1 (25.0%) was confirmed as papillary thyroid microcarcioma (PTMC) (G12 V). Of the 26 nodules positive for *N-RAS* 61, 6 (23.1%) were confirmed as malignant (2PTCs: Q61R, 1PTC: Q61K, 3 FV-PTC: Q61R). The single nodule positive for *H-RAS* 61 was diagnosed as benign on US-FNA cytology. Twenty-four (84.3%) of the nodules positive for *K-RAS* 12/13 (N = 3), *N-RAS* 61 (N = 20), or *H-RAS* 61 (N = 1) mutations were proven benign. None of the 198 thyroid nodules were positive for *K-RAS* 61, *N-RAS* 12/13, or *H-RAS* 12/13 mutations.

**TABLE 1:**
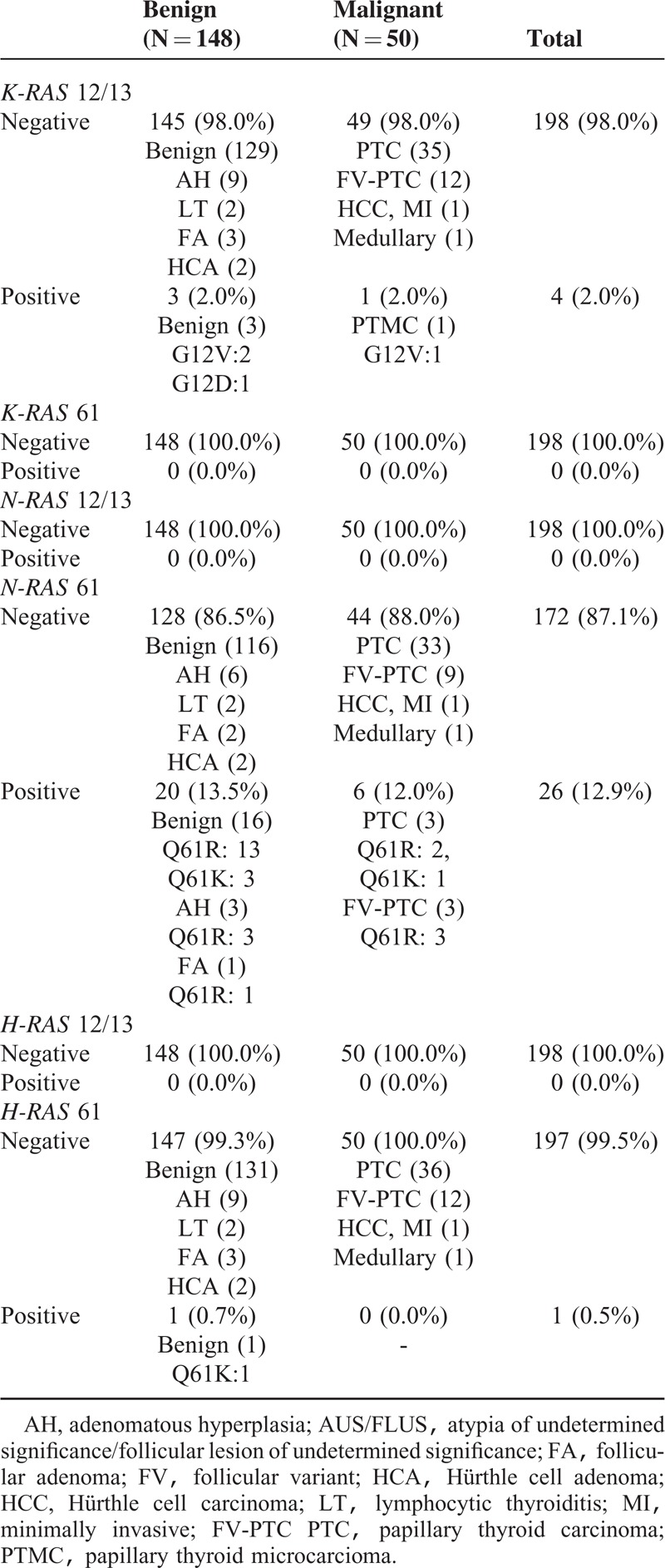
Correlation of *K-RAS*, *N-RAS*, *H-RAS* to Final Pathology Results Among the 198 Thyroid Nodules With AUS/FLUS Cytology and Negative BRAF^V600E^ Mutation

Based on the number of *RAS* mutations present in this study, clinical features among thyroid nodules were analyzed for nodules with any *RAS* mutation (Table 2). Nodule size was significantly larger in nodules positive for *N-RAS* 61 mutation, 20.2 ± 14.5 mm to 14.4 ± 10.4 mm (*P* = 0.01). Patient's age and sex did not show significant differences in thyroid nodules positive for *RAS* mutation (all *P* > 0.05).

**TABLE 2:**
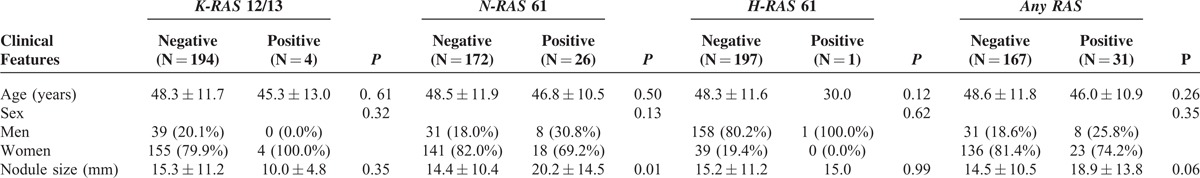
Comparison of Clinical Features Among Thyroid Nodule According to *N-RAS* 61 Mutation

## DISCUSSION

Indeterminate cytology, including AUS/FLUS, has been considered problematic both to the patient and the clinician because a definite diagnosis has not been reached, but still, both the possibility of benignity or malignancy has been suggested with the ambiguous cytologic result. Atypia of undetermined significance/follicular lesion of undetermined significance cytology in particular, has a recommended malignancy rate of 15% to 30% by the Bethesda system,^31^ and although diagnostic lobectomy is recommended in patients with suspicious features for malignancy, the majority of patients with AUS/FLUS cytology is confirmed as benign for which surgery is rather unnecessary. In effort to improve the accuracy of cytological diagnosis, molecular testing has been recently introduced and *BRAF*, *RAS,* and *RET/PTC* mutations have been popularly used and evaluated as an adjunctive tool in detecting thyroid malignancy.^32,33^ BRAF^V600E^ mutation is the most common genetic alteration, which is highly specific for PTC,^5,16,34,35^ has been proven as an useful adjunct to US-FNA cytology in detecting malignancy among thyroid nodules with indeterminate cytology with high positive predictive value ranging from 87% to 100%.^19,33^ But, a considerable proportion of BRAF^V600E^ negative-nodules are proven as malignant, in particular, malignancy rate of BRAF^V600E^ negative-nodules with AUS/FLUS cytology has been reported to be 39.7% to 62.7%.^11,36,37^ Malignancy rate of BRAF^V600E^ mutation-negative nodules with AUS/FLUS cytology in this study was 25.3%, slightly lower than the prior reports, but showing that a considerable amount of thyroid cancers are missed by BRAF^V600E^ mutation analysis alone.

In addition to BRAF^V600E^ mutation, *RAS* mutation, the second most common genetic alteration in thyroid cancer,^38,39^ has been reported to be helpful in detecting follicular-patterned thyroid tumors, especially FV-PTC among thyroid nodules of indeterminate cytology.^28,38–40^ Results of a recent study show that approximately 40.4% (23 of 57) BRAF^V600E^ mutation-negative nodules with AUS/FLUS cytology had *RAS* 61 mutations,^28^ among which 52.2% were surgically confirmed as FV-PTC. The presence of *RAS* mutations suggests a possibility of a broad spectrum of tumors from benign follicular adenoma to follicular thyroid carcinoma, FV-PTC, anaplastic carcinoma and poorly differentiated thyroid carcinoma,^28,38,39,41,42^ conditions that are well known to be difficult to diagnose with cytology alone since they are often diagnosed as indeterminate with FNA, also with the majority negative for BRAF^V600E^ mutation. Most of the studies evaluating the efficacy of *RAS* mutation focus on thyroid nodules with indeterminate cytology, including AUS/FLUS, follicular neoplasm or suspicious for follicular neoplasm, and/or suspicious for malignancy categories,^28,38–40^ and to our knowledge, there are no studies published evaluating the usefulness of *RAS* mutation among AUS/FLUS cytology category alone in detecting thyroid cancers.

In this study, 15.7% (31 of 198) of the BRAF^V600E^ mutation-negative thyroid nodules with AUS/FLUS cytology were positive for *RAS* mutation, of which 22.6% were proven malignant. Also, 42.9% (3 out of 7 malignant nodules) were confirmed as FV-PTC on surgery, the remaining 57.1% diagnosed as PTC. Both the proportion of the presence of *RAS* mutation and the positive predictive value of *RAS* mutation was low, even when considering that this study had exclusively included nodules with AUS/FLUS cytology. Similar to the results of our study, in another study comparing the diagnostic utility of BRAF^V600E^ mutation to *RAS* and *RET/PTC* rearrangements, the presence of *RAS* mutation in AUS/FLUS cytology was 7.7%, whereas cancer risk was 0%.^5^ Unlike BRAF^V600E^ mutation, in which the presence of mutation almost always predicts malignancy, the predictive value of *RAS* mutation is not as confirmative, since *RAS* mutations is also detected in benign follicular adenomas, which obscures the performance of this specific mutation, which must be considered when applying it as an adjunctive diagnostic tool. As the prior study concluded, based on the results of our study, we feel that the presence of *RAS* mutation do not enhance the detection of thyroid cancers BRAF^V600E^ mutation-negative thyroid nodules with AUS/FLUS cytology, even those of follicular patterned growth.

*RAS* mutation consists of 3 highly homologous human *RAS* genes, *N-RAS*, *K-RAS*, and *H-RAS* carrying mutations in codons 12, 13, and 61 have been described to be related to thyroid tumorigenesis.^38,39^ The presence of different subtypes of *RAS* mutation has been reported to be related to different clinicopathological outcomes, *K-RAS* 12/13 mutation showing significantly lower carcinoma outcome compared with *N-RAS* 61 or *H-RAS* 61 mutations.^26^ Our study showed similar results in that *K-RAS* 12/13 mutation has a low malignancy rate, 75% (3 of 4) of nodules with *K-RAS* 12/13 mutation were proven benign. *N-RAS* 61 mutation was the most common mutation (83.9%, 26 of 31), as with the results of previous reports,^26,43^ of which approximately 50% of nodules with *N-RAS* 61 mutation was proven as FV-PTC, consistent to the prior report in that FV-PTC is the cancer subtype mostly related to *RAS* 61 mutation.^26,28,43^ Only 1 benign thyroid nodule had positive results for *H-RAS* 61 mutation, which is in contrast with the prior report showing relatively high cancer rates in nodules with *H-RAS* 61 mutation.^28^ In addition, malignancy rate of *N-RAS* 61 mutation was 23.1% (6 of 26), lower than the 25.0% (1 of 4) for *K-RAS* 12/13, results which are in contrast to the report in that *K-RAS* mutation witholds lower malignancy rate than *N-RAS* mutation.^26^ The small number of nodules positive for mutation may have affected the results, and as little has been proven in the true characteristics of *RAS* mutation subclassification, further studies including a large number of patients are anticipated to evaluate the true utility of *RAS* mutation when applied to lesion characterization or for risk stratification for patient management.

There are several limitations to this study. First, this study is of a retrospective design in which selection bias may have occurred during patient inclusion. Second, only 31.2% of the nodules included in this study had been confirmed with surgery, whereas the remaining 68.8% had been diagnosed based on cytology results. Third, subcategorization of AUS/FLUS cytology has not been applied. Prevalence of *RAS* mutation and predictive value for thyroid malignancy may differ among subcategories of AUS/FLUS which has not been considered in this study. Fourth, 1 of 5 cytopathologists was involved in cytology interpretation, in which interobserver variability may have existed. Fifth, direct sequencing has been used for mutation analysis in this study, a method which has been known to exclude mutations present in minor fractions of tumor cells,^44^ which may have affected our results.

In conclusion, *N-RAS* 61 mutation is the most common mutation detected among BRAF^V600E^ mutation-negative nodules with AUS/FLUS cytology. *RAS* mutation has limited value in predicting malignancy among BRAF^V600E^ mutation-negative thyroid nodules with AUS/FLUS cytology and further investigation is anticipated to evaluate the true role of *RAS* mutation in thyroid malignancy.
